# ECMO for the pregnant and peripartum patient: A practical review of indications, unique management considerations, and an approach framework

**DOI:** 10.1177/02676591251321070

**Published:** 2025-02-23

**Authors:** Carmen S. Hrymak, Ahmed Labib, Bindu Akkanti, Marta V Antonini, Bradley Bruggeman, Matthew J Griffee, Silver Heinsar, Jeffrey P Jacobs, Michelle Larzelere, Emily Naoum, Erika O’Neil, Dikea Roussos-Ross, Akram M Zaaqoq, Giles J Peek, Rakesh C Arora

**Affiliations:** 1 Department of Emergency Medicine and Section of Critical Care, 12359 University of Manitoba, Winnipeg, MB, Canada; 2 Medical Intensive Care Unit, 36977 Hamad General Hospital, Doha, Qatar; 3 Weill Cornell Medicine, Doha, Qatar; 4 Division of Critical Care Medicine and Advanced Cardiopulmonary Therapeutics and Transplantation, 12340 UT Health- Houston, Houston, TX, USA; 5 Bufalini Hospital, AUSL Romagna, Cesena, Italy; 6 Department of Obstetrics and Gynecology, 12233 University of Florida College of Medicine, Gainesville, FL, USA; 7 Department of Anesthesiology, 12348 University of Utah School of Medicine, Salt Lake City, Utah, USA; 8 629337 Critical Care Research Group, The University of Queensland, Brisbane, QLD, Australia; 9 Department of Intensive Care, 91887 North Estonia Medical, Tallinn, Estonia; 10 497729 University of Florida Congenital Heart Center, Gainesville, FL, USA; 11 Department of Anesthesia, Critical Care, and Pain Medicine, 2348 Massachusetts General Hospital, Boston, MA, USA; 12 Department of Pediatrics, United States Air Force, 3998 Brooke Army Medical Center, San Antonio, TX, USA; 13 Department of Anesthesiology, Division of Critical Care, 2358 University of Virginia, Charlottesville, VA, USA; 14 497729 University of Florida Congenital Heart Center, Gainesville, FL, USA; 15 Department of Surgery, Division of Cardiac Surgery, 574761 University Hospitals – Harrington Heart Vascular Institute, Cleveland, OH, USA

**Keywords:** COVID-19, pregnancy/pregnant, ECMO, ARDS, maternal, fetal, amniotic fluid embolism, pre-eclampsia, right ventricular failure, cardiogenic shock, pulmonary embolism, fetal heart rate

## Abstract

The use of extracorporeal membrane oxygenation (ECMO) to support the pregnant patient and fetus requires a complex decision-making process. Peripartum ECMO requires coordinated and informed decision-making between an interdisciplinary team of experts, incorporating the unique considerations and, at times, competing physiologic priorities of the pregnant patient. It is often confounded by a scarcity of local relevant experience engendered by its rare occurrence. No event has made the need for an organized approach to the utilization of ECMO in pregnant patients more pressing than the COVID pandemic. The conditions affecting pregnant patients that warrant ECMO consideration are high stakes and, at times, ethically challenging, although outcomes are favourable compared to the general population. This review provides background information and context, followed by a practical approach to the care and specific medical management of patients who are facing life-threatening conditions warranting ECMO while pregnant.

## Introduction

The use of an approach to extracorporeal membrane oxygenation (ECMO) in special populations, such as pregnant patients, has been under-reported, thus posing a challenge for decision-making in urgent situations. Meanwhile, ECMO has been shown to be safe in well-selected pregnant and peripartum patients.^1,2^

During the 2009 H1N1 influenza pandemic, pregnant patients were more likely to develop complications than nonpregnant patients, and some of the earlier reports of the use of ECMO included their experiences in this population.^3,4^ In addition, comparing pregnant to non-pregnant patients receiving ECMO from 2013 to 2019, a recent systematic review demonstrated lower rates of death, bleeding complications, length of stay, and inpatient cost.^5^ While rates of ECMO in pregnancy have increased over time, it appears to remain relatively under-utilized.^5,6^

The physiologic, hormonal and immunomodulatory changes during pregnancy increase the risk of severe respiratory system involvement due to infection.^7^ While practice advisories from the American College of Obstetricians and Gynecologists^8^ and the CDC have been issued,^9^ there remains limited data on the efficacy of ECMO in these patients. Comparing pregnant to nonpregnant females, among 1180 propensity score-adjusted female patients supported with VV ECMO for COVID-19, the 100 pregnant patients were more likely to survive to hospital discharge (62%) and suffer fewer ECMO-related complications.^10^ In a systematic review of case reports of ECMO in pregnancy, the indication was cardiovascular failure in 61% of the total 97 patients, with the remainder for respiratory failure (ARDS 91.9%, pulmonary embolism 23.7%, and peripartum cardiomyopathy 16.9%).^11^ Survival was high for patients and neonates; however, this literature is subject to publication bias inherent to case reports.^11^ A retrospective observational study of pregnant and peripartum patients requiring ECMO for COVID-19 again supported favourable survival.^12^

Lessons learned in critical care,^13^ the facilitation of shared-decision making processes,^14^ and management of the pregnant patient on ECMO,^15^ continued to evolve. Thus, there is a need to refine the approach to these patients to ensure timely and effective care to maximize favourable outcomes.

This review aims to provide ECMOlogists, physicians with overall responsible for the ECMO patient, and the interprofessional team caring for the pregnant patient with (1) background knowledge relevant and practical to facilitating care decisions and (2) a state-of-the-art approach framework with the overarching goal of knowledge sharing to facilitate communication and decision-making. First, we provide an overview of the physiological and anatomical changes of pregnancy and discuss the indications of ECMO. Next, we focus on the unique considerations of pregnant patients and fetal challenges in ECMO management during pregnancy. Lastly, we provide a practical approach framework to support rapid, interdisciplinary interaction to provide timely shared decision-making for pregnant patients facing high-stakes and often high-emotive scenarios.

## Physiological and anatomical considerations

**Figure 1.**
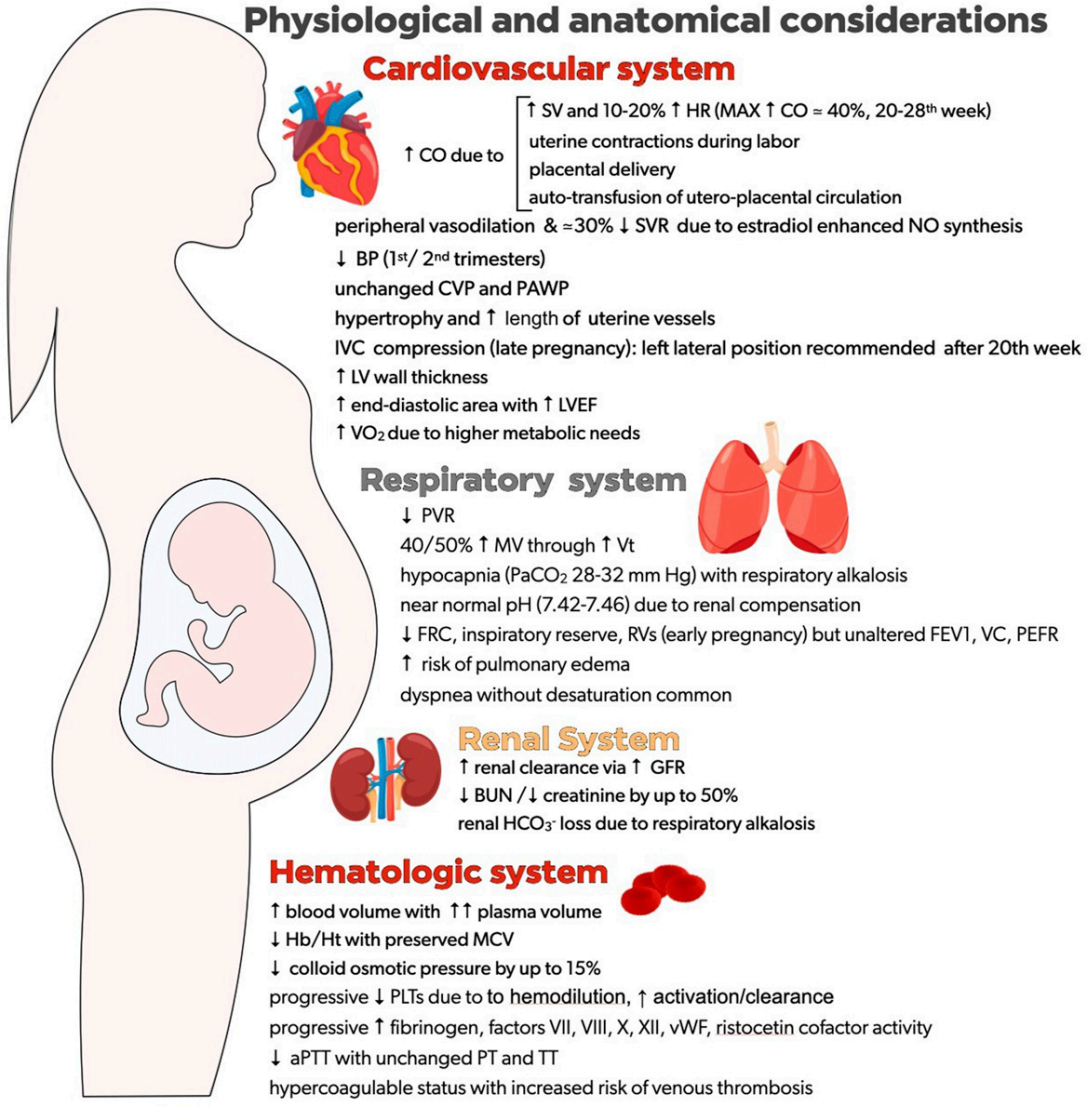
Physiologic and anatomic consideration in pregnancy.

Pregnancy-induced multi-organ physiological changes - brought on by hormonal, metabolic and anatomical factors - prepare the patient for the stress of pregnancy, labour and the immediate postpartum period. Pregnancy impacts almost all body systems, of particular relevance to ECMO are the cardiovascular, respiratory, renal, and hematological systems.^16,17^ These changes, which are most pronounced in the second and third trimester, are summarized in Figure 1.

### Cardiovascular

Adaptive changes to the cardiovascular system during pregnancy and the early postpartum period, including increased blood volume, cardiac output, and decreased peripheral vascular resistance, have significant implications for ECMO. Uterine vessels develop outward hypertrophy and lengthen.^18^ Despite the anticipated increase in vascular resistance, decreased vascular resistance occurs due to angiogenesis, which ensures the delivery of nutrients and oxygen to the fetus.^19^ Estradiol enhances nitric oxide synthesis, causing peripheral vasodilation and a drop in systemic vascular resistance by almost 30%. A compensatory adaptive mechanism of increased cardiac output (CO) starts as early as the 8^th^ week of gestation.^20^ Augmented CO is achieved mainly via increased stroke volume and some 10–20% increase in the heart rate. The maximum increase in CO of around 40% is typically seen around the 20–28^th^ week of gestation. Another increase in CO is observed during labor due to uterine contractions and a further increase follows placental delivery and “auto-transfusion” from the utero-placental circulation.^21,22^ Gravid uterus compression of the inferior vena cava can lead to hemodynamic compromise and left lateral position after the 20^th^ week of gestation is recommended to maintain utero-placental flow.^21^ Aside from compression of the aorta and the inferior vena cava by the gravid uterus in late pregnancy, pregnancy has a limited impact on the major pelvic blood vessels.

Echocardiographic findings include an increased left ventricular (LV) myocardial wall thickness and end-diastolic but not an end-systolic area. This translates into increased LV ejection fraction.^23,24^ Blood pressure, affected mainly by systemic vascular resistance, falls in the 1^st^ and 2^nd^ trimesters, then returns to pre-pregnancy level in the 3^rd^ trimester. The complex effect of intravascular and stroke volume does not translate into an equivalent rise in filling pressures: both central venous and pulmonary capillary wedge pressure do not increase. An increased circulatory volume with preserved myocardial contractility and relaxation are cardinal features of a healthy pregnancy.^25^

### Respiratory

Colloid osmotic pressure is reduced by up to 15%, and pulmonary vascular resistance is decreased. Pregnant patients are susceptible to both cardiogenic and non-cardiogenic pulmonary edema, particularly in conditions associated with increased preload or pulmonary blood flow, such as left heart failure or the capillary leak associated with ARDS.^26^

Pregnancy induces increased oxygen demand due to a higher metabolic need (pregnant patient and fetus) and increased oxygen consumption. Remarkably, a higher tidal volume, rather than an increase in respiratory rate, leads to a 40–50% rise in minute ventilation. This translates into respiratory alkalosis and renal compensatory bicarbonate loss tends to maintain a slightly alkaline pH of 7.42-7.46. Typically, a pregnant arterial blood gas analysis value of PaCO_2_ 28–32 mmHg is anticipated, and a “*normal*” PaCO_2_ of 40 mmHg should alert the physician to respiratory compromise.^27^ Functional residual capacity, inspiratory reserve, and residual volumes are reduced early in pregnancy, whereas vital capacity, peak expiratory flow rate and forced expiratory volume in one second remain unaltered. Dyspnea without desaturation is common during pregnancy.^28,29^

### Renal

Renal clearance is enhanced via increased glomerular filtration rate, reducing blood urea nitrogen and serum creatinine by up to 50% compared to pre-pregnancy values.^30^

### Hematologic

Plasma volume is expanded with less of an increase in red cell mass, leading to physiological anemia of pregnancy. Reduced hemoglobin and hematocrit, with preserved mean corpuscular volume, are typical changes in pregnancy. As pregnancy progresses, there is an increase in fibrinogen, factors VII, VIII, X, XII, von Willebrand factor, and ristocetin cofactor activity. The shortening of activated partial thromboplastin time reflects the increase in coagulation factors,^31^ yet prothrombin time (PT) and Thrombin time remain similar.^32^ Gestational thrombocytopenia is marked by a progressive decline in platelet count attributable to hemodilution, increased platelet activation, and accelerated clearance.^27,33,34^ Overall, a hypercoagulable status prepares for hemostasis following delivery but also increases the risk of venous thrombosis.

## Indications for VV and VA ECMO in pregnancy

Common indications for VV and VA ECMO are listed in Panel 1. It is important to appreciate that either VA or VV ECMO can be used to support pregnant patients who are deteriorating despite optimal conventional management. In the context of a severely compromised pregnant patient, the increase in either oxygen delivery, blood flow, or both following ECMO initiation results in increased fetal oxygen delivery without increased risk of spontaneous fetal loss or intra-uterine catastrophe.

With respect to candidacy, pregnant patients with acute cardiorespiratory failure may be better candidates for ECMO when compared to older patients with ischemic heart disease and decompensated, long-standing cardiomyopathy - a population commonly considered for mechanical circulatory support.^35^ Outcomes may be better for patients who require ECMO in the antepartum period or at the time of delivery, compared to outcomes for patients treated with VA ECMO for cardiogenic shock presenting post-partum.^36,37^ The indication for VA ECMO in peripartum women with the highest reported survival is for cardiac arrest, with 87.7% survival, and neurologically intact survival of 78.9%.^36^ Limited data suggest that early cannulation (within 5 days of mechanical ventilation) is associated with better outcomes than later cannulation.^37,38^

Intensivists, surgeons, and maternal medicine specialists should be aware that VA ECMO in the peripartum period has superior outcomes to outcomes seen for the general population. Tables 1 and 2 summarize current evidence for ECMO in pregnant patients.^36^

**Table 1.** Systematic reviews and recent case series of peripartum VV ECMO.^36,37,39–45^

Study type Lead author Year	Number of VV ECMO cases, fraction total ECMO cases	Indication for VV ECMO	Complication summary	Maternal survival	Fetal survival
Case series Wong RW 2024	11/18(61%) Up to 6 weeks post-partum	COVID-19 Other pulmonary	Infections 24% Oxygenator failure 13%	Overall survival 78%	Most deliveries performed before ECMO cannulation
Case series Kakar V 2024	5/5	COVID-19 ARDS	Critical illness myopathy (1); barotrauma (2)	100%	3/5
Case series Li Y 2022	2/5	Postpartum hemorrhage; aspiration and shock	Not described	100%	2/2
Systematic review Naoum EE 2020	96/358 (27%)	ARDS Asthma Cardiac failure	Mild-mod bleeding (30%); severe bleeding (12%)	80%	Not specified
Systematic review Sebastian NA 2022	86/213 (40%)	Not specified specifically for VV ECMO	Not specified	Overall survival 79%	Not specified
International registry Malfertheiner SF 2023	46/60 (77%)	ARDS from influenza; pneumonia	Not reported in detail, see page 5 of paper	94%	Not specified
Case series Lankford AS 2021	13/21 (62%)	ARDS (12), cystic fibrosis (1)	Bleeding (6)	77%	100% (incomplete follow up, 6/6 infants with data)
Multicenter registry Hao X 2023	15/37 (41%)	Not specified VV: severe pneumonia Cardiac arrest Pulmonary arterial hypertension	Hemorrhagic events (30%)	80%	41% overall; 77.8% in third trimester – not specified VV
Systematic review and meta-analysis Shamshirsaz AA 2024	386 total ECMO	Critical COVID-19 related illness	Venous thromboembolism 37.1% Acute kidney injury 19.9% Cardiac complications 17.8% Ischemic stroke or ICH 7.4%	75.6% (similar across trimesters)	83.7% (128/153)

Abbreviations: ARDS - acute respiratory distress syndrome, ICH – intracranial hemorrhage

**Table 2.** Systematic reviews and recent case series of peripartum VA ECMO from ECMO referral centers.^36,37,39,40,43–48^

Type of study Lead author Year	Number of VA ECMO cases, fraction total ECMO cases	Top 3 indications	Complication summary	Maternal survival	Fetal survival
Case series Wong RW 2024	7/18 (39%) Up to 6 weeks post-partum	COVID-19 Peripartum CM	Infections 24% Oxygenator failure 13%	Overall survival 78%	Most deliveries performed before ECMO cannulation
Systematic review Naoum EE 2020	145/358 (40.5%)	Cardiac arrest Cardiac failure Peripartum CM cardiomyopathy	Mild-mod bleeding 20% Severe bleeding 15.9% Brain injury 6.9%	72.4% (105/145)	64.7% overall- not specified for VA
Systematic review Sebastian NA 2022	91/213 (42.7%)	ARDS PE Peripartum CM	Not reported	79.3% (169/213)- not specified for VA	73.7% overall- not specified for VA
International registry Malfertheiner SF 2023	14/60 (23.3%)	PE Peripartum CM Hemorrhage	Major bleeding 23% Ischemic event 23% (PE, DVT, ischemic colitis, arterial clot)	71% (10/14)	52.9% (9/17)
Case series Lankford AS 2021	8/21 (38.1%)	Heart failure (septic cardiomyopathy, peripartum cardiomyopathy) PE	Major bleeding 33% PE (n=2) Limb ischemia (n=2)	62.5% (5/8)- one required cardiac transplant	100% (4/4) – other infant data not available
Case series Desai M 2022	7/7 (100%- VA ECMO series)	Peripartum cardiomyopathy Septic CM Acute RV failure	Bleeding (2 had interventions); one brain injury	71.4% (5/7)	83.3% (5/6)
Case series Aissi James 2022	10/10	Amniotic fluid embolism (AFE series, 100%)	Acute Kidney (80%) Acute Liver (70%)	70% (7/10)	100% (10/10)
Multicenter Registry Hao X 2023	22/37 (59%)	Not specified VA Severe pneumonia Cardiac arrest Pulmonary arterial hypertension	Hemorrhagic events 30%	56% (9/16) – VA 17% (1/6) – ECPR	41% overall; 77.8% in third trimester – not specified VA
Case series Buda, Kevin 2024	34/34	Amniotic fluid embolism (AFE series, 100%)	Renal replacement (29%) Brain death (9%)	62% (21/34)	N/A
Systematic review and meta-analysis Shamshirsaz AA 2024	386 total ECMO	Critical COVID-19 related illness	Venous thromboembolism 37.1% Acute kidney injury 19.9% Cardiac complications 17.8% Ischemic stroke or ICH 7.4%	75.6% (similar across trimesters)	83.7% (128/153)

Abbreviations: CM – cardiomyopathy, DVT – deep venous thrombosis, ICH – intracranial hemorrhage, PE – pulmonary embolism, RV – right ventricle

## Panel 1: Indications for ECMO

See Figure 2

**Figure 2.**
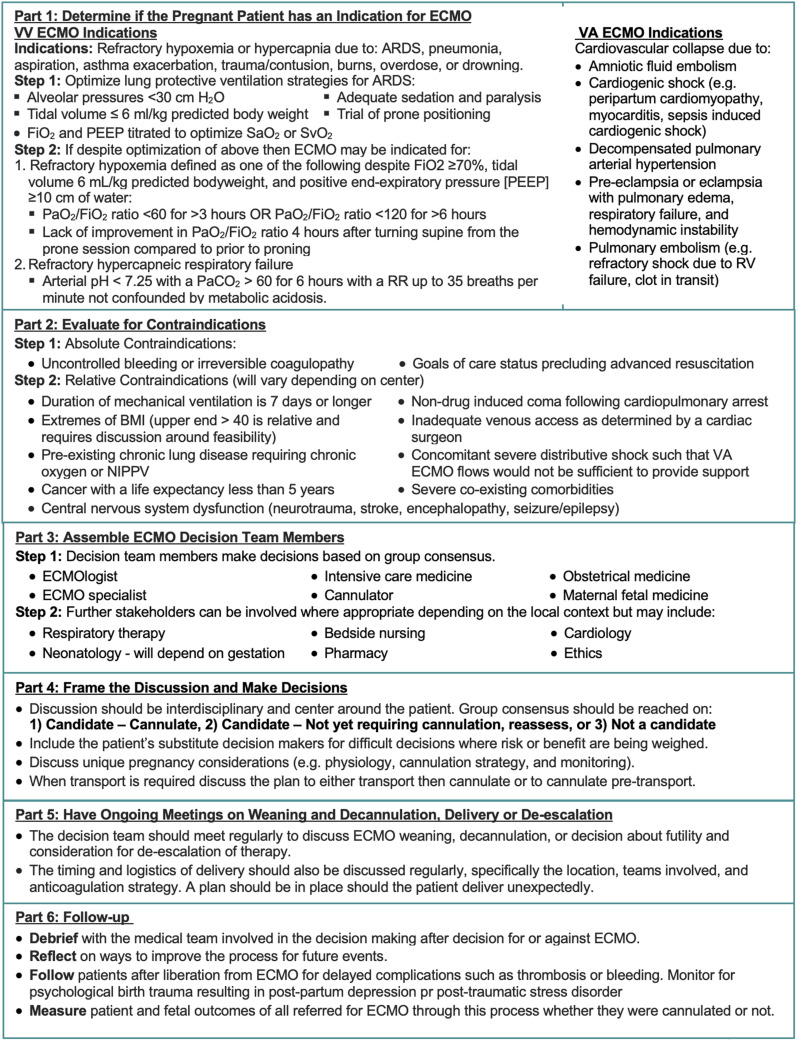
Approach framework for ECMO referral in pregnancy.

***VV ECMO indications***

Refractory hypoxemia or hypercapnia due to:

 ⁃ ARDS
 ⁃ Pneumonia
 ⁃ Aspiration
 ⁃ Asthma exacerbation
 ⁃ Trauma/contusion
 ⁃ Burns
 ⁃ Overdose
 ⁃ Drowning

**Step 1:** Optimize lung protective ventilation strategies for ARDS (see Figure 2)

 ⁃ Alveolar pressures <30 cm H_2_O
 ⁃ Adequate sedation
 ⁃ Tidal volume ≤6 mL/kg predicted body weight
 ⁃ Trial of prone positioning
 ⁃ Paralysis
 ⁃ FiO_2_ and PEEP titrated to optimize SaO_2_ or SvO_2_

**Step 2**: If despite optimization of above then ECMO may be indicated for:

1. Refractory hypoxemia defined as one of the following despite FiO_2_ ≥70%, tidal volume 6 mL/kg predicted bodyweight, and positive end-expiratory pressure [PEEP] ≥10 cm of water:
  ⁃ PaO_2_/FiO_2_ ratio <60 for >3 hours OR PaO_2_/FiO_2_ ratio <120 for >6 hours
  ⁃ Lack of improvement in PaO_2_/FiO_2_ ratio 4 hours after turning supine from the prone session compared to prior to proning
2. Refractory hypercapneic respiratory failure
  ⁃ Arterial pH < 7.25 with a PaCO_2_ > 60 for 6 hours with a RR up to 35 breaths per minute not confounded by metabolic acidosis

***VA ECMO Indications***

Cardiovascular collapse due to:

⁃ Amniotic fluid embolism
⁃ Cardiogenic shock (e.g. peripartum cardiomyopathy, myocarditis, sepsis-induced cardiogenic shock^49^)
⁃ Decompensated pulmonary arterial hypertension or RV failure from pulmonary arterial hypertension
⁃ Pre-eclampsia or eclampsia with pulmonary edema, respiratory failure, and hemodynamic instability
⁃ Pulmonary embolism (e.g. refractory shock due to RV failure, clot in transit)

## ECMO cannulation

The urgency influences the selection of the cannulation strategy during pregnancy as well as the type of required support, access to the blood vessels, and operator skills. One of the challenges during pregnancy is access to the femoral blood vessels, especially in morbidly obese patients.^50^ Hence, the use of standard imaging techniques such as ultrasound or fluoroscopy is recommended.^51^ The risk of radiation exposure to the fetus or breast tissue compared to the benefit of fluoroscopic imaging has to be considered on an individual basis. Another challenge is advancing the guidewire and the cannula into the inferior vena cava that the gravid uterus may be compressing. Ngatchou and colleagues recommended a left tilt position during femoral cannulation to prevent aortocaval compression by the gravid uterus and facilitate advancing the guidewire and the venous cannula.^52^ A wedge-shaped cushion, rolled blanket, or pillow can achieve a 15-30-degree left tilt. The use of jugular dual-lumen venovenous cannulation avoids this issue. Some pregnant patients may have May-Thurner syndrome due to compression of the right common iliac artery against the fifth lumbar vertebra, causing left common iliac vein obstruction and thrombosis, increasing the risk of deep venous thrombosis. Cannulation strategy may need to take this into account in difficult scenarios.^53^

## VV ECMO specifics

### Configuration

A dual-lumen cannula has the advantage of a single access site (usually the right internal jugular vein), which frees other venous access sites and might promote mobility and physical therapy.^50^ Another increasingly popular form of support is venopulmonary ECMO, which uses either a dual-lumen cannula or two single-lumen cannulas to drain from the right atrium and return to the pulmonary artery. The pulmonary artery configuration has the advantage of bypassing the right ventricle, making it ideal support if right ventricular failure arises from increased PVR of severe ARDS. In a multi-center study comparing different VV ECMO configurations in 435 patients with COVID-19-associated severe ARDS, the venopulmonary configuration was associated with the lowest mortality at 90 days; however, this was an observational study and further research is needed.^54^

On the other hand, two-site or multisite VV cannulation may enhance total ECMO flow, which would be required in a higher cardiac output state such as pregnancy.^50,55^ All cannulation strategies must be coupled with adequate placement confirmation and avoidance of recirculation.

### Management

The described changes in physiology and anatomy affect not only the approach of cannulation but also the day-to-day management of the pregnant patient on VV ECMO. Regarding the gravid uterus, the challenge is overcoming compressive effects. Increased abdominal pressures on lung structures may require rest settings with a higher PEEP strategy to avoid excessive loss of lung volume on expiration. The driving pressure should be limited to 10 cm H_2_O, as in any patient on ECMO, and an esophageal balloon can assist in titration settings to optimize and avoid lung injury.^56^ The uterus also compresses vascular structures, so tilting the patient to the left with a wedge or pillows is necessary, with meticulous skin care to avoid pressure wounds. Based on the high CO in pregnancy, there may be a need to rely on the lungs in part for oxygenation, depending on the amount of VV ECMO flow present. If lung protective ventilation is not achievable, steps should be taken to increase extracorporeal flow and oxygen delivery, such as adding an extra drainage or return cannula. We recommend a trial of proning before initiation of VV ECMO. is an evidence-based strategy for ARDS management. Although this has been employed in pregnant patients on VV ECMO, a recent randomized clinical trial on non-pregnant VV ECMO patients failed to show significant benefit.^57^ Lateral decubitus position has also been described in patients who cannot tolerate proning.^58^

### Anticoagulation during ECMO

Anticoagulants are frequently used during ECMO to prevent clotting of the circuit or membrane. Pregnancy is a hypercoagulable state, and pregnancy-related complications are associated with an increased risk of thrombosis among patients receiving ECMO, making anticoagulation an especially important topic.^59^ Intravenous heparin titrated to an aPTT goal per an ECMO-specific protocol has been the standard anticoagulant during ECMO thus far and has been used safely during pregnancy, as it does not cross the placenta. Direct thrombin inhibitors argatroban and bivalirudin have become increasingly popular, replacing heparin in many units. Both agents have been used successfully during ECMO in the third trimester of pregnancy in a limited number of patients.^57,60–62^ There is currently insufficient evidence to either recommend or advise against the use of direct thrombin inhibitors during ECMO support of pregnant patients.

The major complications seen in pregnant and postpartum women requiring ECMO include hemorrhage, thromboembolic events, and vascular complications.^36^ However, the rates were similar to non-pregnant patients requiring ECMO.^63,64^ Risk versus benefit considerations of anticoagulation therapy to mitigate thromboembolic events in a patient with an acute bleed may require discontinuation.

Second-generation heparin-coated circuits decrease the need for anticoagulation, and recent publications suggest no anticoagulation may be appropriate in certain circumstances such as amniotic fluid embolism, where the risk of bleeding would historically have precluded this indication.^46^

## Delivery considerations

Delivery considerations are outlined in Table 3 in a way that emphasizes the potential benefits, lack of benefits, or harms of delivery.

**Table 3.** Delivery considerations.

Delivery consideration	Potential benefits or harms of delivery
Obstetric complications requiring delivery^65^	• Fetal delivery may be required for obstetrical reasons^66^ e.g. preeclampsia, eclampsia, HELLP syndrome, fetal growth retardation, and fetal distress
Delivery based on worsening respiratory status	• Delivery decreases oxygen requirements up to 28%^67^
	• Delivery does not decrease duration of either mechanical ventilation or ECMO cannulation^68^
Delivery prior to ECMO cannulation versus after cannulation	• Delivery increases the risk of postpartum hemorrhage but there is no statistical difference in rates of transfusion^67^
	• Data from pregnant patients with acute ARDS have indicated that there is an increased risk of fetal loss after cannulation, however delay may decrease the risk of prematurity-related morbidity in the newborn^68^
	• There is no difference in rate of other obstetric complications such as preeclampsia or gestational diabetes^68^
Delivery prior to viability	• There is no evidence that terminating a pregnancy for respiratory indications prior to the third trimester will improve the outcome of the pregnant patient^67^
Delivery >28 week gestation or at term	• Mechanical respiratory changes from pregnancy including upward displacement of the diaphragm and decreased end expiratory reserve volume coupled with the burden of the fetoplacental unit on oxygen consumption may shift the risk-benefit towards delivery^67,69,70^
Route of delivery	• Cesarean delivery, induced vaginal delivery, and spontaneous expulsion of the neonate have been described without specific benefit of one over the other^36,71^

### Indications for delivery

There are no expert consensus statements or current universal guidelines for the optimal timing of delivery in critically ill pregnant patients who require ECMO. Vaginal, assisted-vaginal, and cesarean deliveries have been described in the case series, and the mode of delivery should be determined by indications.^15,36,37^ In general, prolonging pregnancy is in the best interest of the fetus, and delivery prior to 30–32 weeks is unlikely to confer significant pulmonary benefit to the mother.^39,64,69^ The potential hemodynamic stress related to labor, cesarean delivery, bleeding, and the immunologic risks to the pregnant patient during delivery, must be balanced with the perceived benefit of delivering the fetus. ECMO support is not an indication for delivery; however, in the setting of an ongoing pregnancy while on extracorporeal support there must be interdisciplinary involvement in care and development of a plan for delivery if required.^72,73^ Delivery route and the location of delivery regarding delivery should be based on an interdisciplinary approach. Factors to consider include gestational age and potential benefits to the newborn, comorbidities of the pregnant patient, progression of the current illness, and local provider expertise.^36,69,71^

The most common indications for delivery in those with a COVID-19 infection included preeclampsia/eclampsia/HELLP syndrome, fetal growth retardation (FGR), and fetal distress. Rates of premature rupture of membranes (PROM) were similar between those with an acute infection and those without.^74^ When these conditions are diagnosed without other changes in maternal status, delivery timing can be recommended based on indication and gestational age.^66^

Clinical deterioration of the pregnant patient should prompt an interdisciplinary discussion regarding the risks and benefits of continuing the pregnancy and have shared decision-making with the patient’s family and/or healthcare proxy.^69,75^

### Perimorten cesarean delivery

For patients who experience cardiac arrest, the American Heart Association^76^ and other international societies^77,78^ have published guidelines regarding the role of perimortem cesarean delivery (PMCD). If return of spontaneous circulation (ROSC) cannot be established by 4 minutes from the time of arrest, PMCD should be considered in patients for whom the uterine fundus is at or above the umbilicus (corresponding to approximately 20 weeks’ gestational age), with the goal to effect delivery of the fetus at 5 minutes after ACLS. The rationale for PMCD is based on case series showing PMCD resulted in ROSC or improvement in maternal hemodynamic status by emptying the uterus and improving caval compression; in combination with the understanding that neurological damage begins to develop within 4-6 minutes of cardiac arrest.^72^

### Complications of delivery

When the decision to deliver the patient on ECMO is made, providers must prepare for potential complications. The risk of hemorrhagic complications is higher in peripartum patients than in the general population. It is multifactorial, including the need for anticoagulation as well as the delivery itself, whether it is vaginal, assisted vaginal, or surgical.^6,36,73^ These patients frequently require blood transfusions and may have bleeding related to the cannulas, tracheostomy, abdomen, and uterus.^79^

Despite the increased risk of hemorrhage, the mortality risk is not increased compared to the non-pregnant population. The team should consider that ECMO in pregnant versus non-pregnant patients has been associated with lower rates of in-hospital all-cause mortality, albeit in small series.^6,73^ Teams should make clinical preparations for obstetric bleeding, including frequent evaluation for appropriateness of postpartum vaginal bleeding. Management may include large-bore intravenous access, uterine fundal massage, uterotonic medications, adequate blood products for resuscitation, intrauterine balloon tamponade devices, and interventional radiology consultation for possible uterine artery embolization. In the event of massive transfusion being required, the ECMO circuit can be used to administer and warm large quantities of blood directly.

## Fetal considerations

In essence, fetal monitoring is an assessment of fetal oxygenation, allowing for the identification and potential delivery of fetuses at risk for hypoxic injury. Fetal well-being is monitored through the interpretation of electronic fetal heart rate (FHR), specifically by evaluating baseline heart rate, variability, and the presence/absence of accelerations and/or decelerations. A normal baseline FHR is between 110 and 160 beats per minute over at least a 10-min period, with brady- and/or tachycardia suggesting possible acidemia. Moderate variability (defined as fluctuation amplitude 6–25 bpm from baseline) is considered normal, with absent, minimal, or marked variability considered abnormal. Accelerations above the baseline rate are indications of fetal well-being, whereas decelerations below the baseline can suggest fetal acidemia. There are morphologic differences in decelerations associated with different phenomena that can lead to acid-base disturbance (e.g., late decelerations are associated with uteroplacental insufficiency).

Continuous FHR monitoring may be utilized in patients at and beyond the gestational age of fetal viability (22–24 weeks gestational age, depending on institutional neonatal intensive care unit (NICU) variations). The Society for Maternal-Fetal Medicine recommends FHR monitoring when fetal intervention - including delivery - would be considered based on gestational age, fetal and maternal status, and maternal preferences.^69^

In routine obstetric care, there are well-established FHR patterns that would prompt consideration of delivery depending on gestational age. However, in critically ill patients, there is no standard threshold with regards to fetal status for delivery. The simultaneous consideration of maternal status complicates such decisions, and one must consider and anticipate potentially conflicting maternal and fetal interests.

As in normal obstetric settings, FHR monitoring has utility in guiding resuscitative efforts. In response to non-reassuring FHR tracings due to hypovolemia, hypoxemia and hypercarbia, ECMO parameters can be titrated to optimize placental perfusion indirectly.

### Breastfeeding and bonding

Few official guidelines exist for breastfeeding and maternal-infant bonding during a critical illness hospitalization, and none exist for patients on ECMO.^65^ ECMO alone, however, does not prohibit skin-to-skin bonding nor breastfeeding especially if the patient is awake and able to sit and do physiotherapy. According to the Academy of Breastfeeding Medicine, direct breastfeeding is preferred, and efforts to support lactation in hospitalized parents, such as pumping equipment, adequate nutrition, and maximizing the time the infant and mother spend together, should be made. According to the American Society of Hematology and the European Society of Cardiology, breastfeeding while on anticoagulation with heparin is safe.^80^ No recommendations discuss breastfeeding while on intravenous direct-thrombin inhibitors; however, both these societies recommend against breastfeeding while on oral direct-thrombin inhibitors. All medications the breastfeeding patient on ECMO receives should be evaluated for infant safety during breastfeeding.

## Ethical considerations

Perhaps the most unique feature of the critically ill pregnant patient population is that care involves the well-being of the pregnant patient and the fetus. These scenarios challenge patients, their families, and the healthcare providers involved as they introduce ethical dilemmas for which there are no right or wrong answers. Pregnant patients receiving ECMO require care that addresses the physiologic needs of the patient and fetus. Fortunately, for most cases, interventions that improve the well-being of the pregnant patient will also help the fetus. There may, however, be situations in which maintaining the pregnancy is deemed life-threatening or not in the best interest of the patient’s health, and delivery at a pre-viable gestational age may be medically indicated to reduce the maternal risk of morbidity and mortality.^81^ Other difficult situations may include a viable pregnancy but a patient who is too unstable to perform a safe emergent delivery for fetal indications without risking the life of the patient. The balance of management requires thoughtful consideration of the ethical principles, including patient autonomy, beneficence, and non-maleficence, and input from all of the interprofessional team caring for these patients.^75,82^ Consultation with an ethics committee may provide a perspective for patients, families, and caregivers to guide them through complicated clinical cases in this population.

## Decannulation considerations

Following delivery or resolution of the underlying pathology that prompted ECMO, weaning trials should be attempted in the standard way for VV or VA ECMO. Standard criteria for decannulation should be used. Given the hypercoagulable status and high risk of deep venous thrombosis (DVT) with ECMO cannulation, Doppler or ultrasound examination should be done for the lower limbs, inferior vena cava, and IJV (in case of IJV cannulation) 24–72 hours post decannulation and repeated if indicated. Maintaining therapeutic anticoagulation should be considered during this time period until DVTs are ruled out. In addition, the avoidance of venous compression during vein decannulation and the use of a simple skin suture may prevent local thrombus formation.^83^

## Approach framework for ECMO referral in pregnancy

Given the aforementioned complexities of ECMO in pregnancy, a structured and systematic approach is required for decision-making and communication. Figure 2 outlines an approach framework for ECMO referral in pregnancy. The framework is meant to be used in conjunction with the other more detailed information in this paper to inform decision-making. We recommend early assembly of an ECMO Decision Team comprised of an ECMOlogist, ECMO specialist, cannulator, intensive care medicine, maternal fetal medicine, and obstetrical medicine (Figure 2). Further stakeholders can be involved where appropriate and the patient’s substitute decision maker should be included for difficult decisions where risks and benefits are being weighed. This ECMO Decision Team should meet early in the patient’s course prior to significant deterioration to frame the discussion and make decisions about candidacy, cannulation timing, and transport, based on group consensus as described in Part 4 of Figure 2. Of note, for respiratory conditions such as ARDS, there is usually time to determine if conventional therapy has failed before deciding for or against VV ECMO. Due to this fact, suggested cutoffs for VV ECMO consideration in this population have been included in Figure 2. Less decision-making time is usually available in cardiovascular collapse conditions but assembling a team is still necessary before proceeding. We recommend this ECMO Decision Team meet regularly to discuss weaning, decannulation (or in rare cases conversion from VV to VA ECMO), delivery, or de-escalation, as outlined in Part 5 of Figure 2. Follow-up after decision and measurement of patient and fetal outcomes are recommended whether the patient was cannulated or not. Attention to the risk of post-partum depression and need for ongoing psychosocial support is an important part of care that can be emphasized at this time (Figure 2).

## Future needs

As cardiac disease is the leading cause of maternal morbidity and considering the rapidly increasing use of ECMO for pregnant patients, a dedicated registry of indications, complications, and outcomes of peripartum ECMO is needed.^36^ Our interprofessional group suggests that the ELSO registry is the best framework for this registry. The current ELSO case report forms should be expanded to include clinical details of pregnant women who require extracorporeal life support. More research is also needed regarding long-term morbidity, functional status, and the potential impact of cardiopulmonary and psychosocial rehabilitation for peripartum survivors of ECMO. Additionally, there is a need for guidance on specific issues such as breastfeeding and bonding while on ECMO. Finally, post-ECMO care guidelines would be of benefit.

## Appendix

### Abbreviations and definitions

ARDS: Acute respiratory distress syndrome
Cannulator: The person responsible for ECMO cannulation
CDC: Centers for disease control and prevention
CO: Cardiac output
COVID-19: Coronavirus disease −2019
ECMO: Extracorporeal membrane oxygenation
ECMOlogists: Physician who specializes in managing ECMO and directs the care of a patient’s ECMO treatment from start to finish. This physician may be an intensivist, pulmonologist, cardiologist, or interventional cardiologist.
ECMO specialist: Manages ECMO at the patient’s bedside. This role can be filled by a registered nurse, respiratory therapist, or perfusionist with special training.
ELSO: Extracorporeal life support organization
FHR: Fetal heart rate
PMCD: Perimortem cesarean delivery
PPROM: Premature rupture of membrane
ROSC: Return of spontaneous circulation
STS: Society of thoracic surgery
VA ECMO: Venoarterial extracorporeal membrane oxygenation
VV ECMO: Venovenous extracorporeal membrane oxygenation.
